# Isolation, Characterization, Pharmacology and Biopolymer Applications of Licorice Polysaccharides: Review

**DOI:** 10.3390/ma15103654

**Published:** 2022-05-20

**Authors:** Noor Ul Ain, Shuye Wu, Xiang Li, Duxin Li, Zhenqing Zhang

**Affiliations:** College of Pharmaceutical Sciences, Soochow University, Suzhou 215021, China; 20217226002@stu.suda.edu.cn (N.U.A.); wsyleaf01180901@163.com (S.W.); 20205226058@stu.suda.edu.cn (X.L.)

**Keywords:** *Glycyrrhiza*, licorice, polysaccharides, extraction method, structure, bio-activity, modern formulation, biomaterial

## Abstract

Licorice is known as “Gan-Cao” in traditional Chinese Medicine (TCM), belonging to the genus *Glycyrrhiza* (Family: Fabaceae/Leguminosae). It has a long medicinal history and wide applications in China. Polysaccharides of licorice (LPs) are one of the key bioactive components. As herbal polysaccharides attracted increasing interest in the past several decades, their extraction, isolation, structural characterization, pharmacological activities, and medicinal application have been explored extensively. It is worth heeding that the method of extraction and purification effects LPs, apart from specie and origin specificity. This review evaluates the method of extraction and purification and demonstrates its performance in gaining specific composition and its structure-activity relationship, which might lead the readers to a fresh horizon for developing advanced treatment strategies. It is recently reported that the conformation of LPs plays a vital role as biopolymers, such as selenized modification, microencapsulation, nanocomposite, liposome formulation, drug/hydrogel combinations, biosensor device, and synergistic effect with a vaccine. In addition, LPs showed a good thermodynamics profile, as these properties enable them to interact with additional supramolecular interaction by chemical modifications or copolymerization. Functional polymers that are responsive to various external stimuli, such as physical, chemical, and biological signals, are a promising study topic. Thus, LPs are emerging as a new biomaterial that can enhance intended formulation along exerting its inherent medicinal effects. It is hoped that this review will provide a basis for the utilization and further developments of licorice polysaccharides in the vast medium.

## 1. Introduction

Licorice, known as Gan-Cao in Chinese and Liquiritiae radix in Latin, is one of the most widely used herbs around the world. Root and rhizome from dried licorice are one of the world’s oldest and most widely used herbal remedies. It is said that 9/10 TCM formulations contain licorice according to classical TCM theory. Due to its sweet taste (up to 150 times sweeter than sugar [1]), it is used as candy, a flavoring agent in cooking, and in tobacco. Licorice belongs to the genus *Glycyrrhiza*, family “Leguminosae/Fabaceae”, containing 29 species and 6 variations worldwide. Three of them are legitimately verified TCM plants, namely *Glycyrrhiza uralensis* Fisch, *G. glabra* Licorice*,* and *G. inflata* Batalin, and they are prescribed as licorice (Hereinafter referred to as *ural* licorice, *glabra* licorice, and *inflata* licorice) [2,3,4].

Licorice is a small shrub with oval leaflets, white or purplish flower clusters, flat pods, a main taproot, and several runners, and it is mostly growing in arid and semi-arid desert grassland, desert edge, and loess hilly areas. Ethnopharmacological studies have demonstrated that licorice can stimulate energy, clear heat, detoxify the body, lubricate the lungs and relieve congestion, ease spasm, and discomfort, and minimize drug adverse effects [4,5,6,7,8]. Over 300 distinct chemicals are found in licorice, some of which have antibacterial, antiviral activities, antitumor, anti-inflammatory, anti-diabetic, and hepatoprotective, etc. [9,10,11,12,13,14,15]. The main chemical constituents are triterpenoid saponins, flavonoids, alkaloids, coumarins, polysaccharides, and proteins [4,15,16]. The small molecules in licorice have been reviewed previously [17,18,19,20,21].

**Figure 1:**
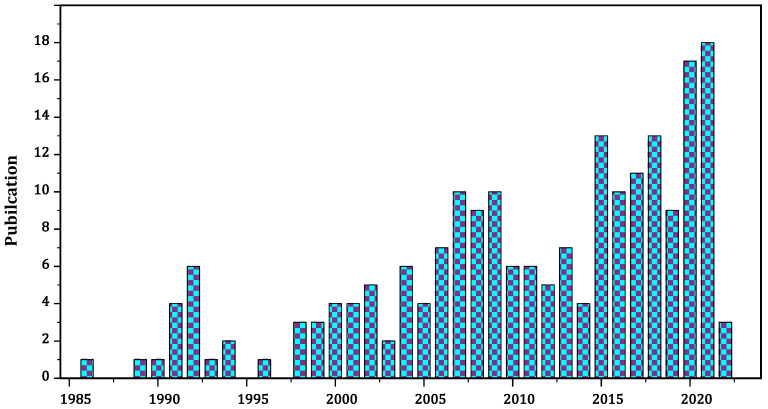
Number of research publications based on licorice polysaccharides.

Polysaccharides have also caught researchers’ interest due to their unique properties, such as non-toxicity and non-specific immune system stimulants [22,23,24]. Its extraction, isolation, structural characterization, pharmacological activities, and medicinal applications have been explored extensively. Data obtained from “China National Knowledge Infrastructure” https://oversea.cnki.net/index/ (accessed on 20 February 2022) and “PubMed.gov” https://pubmed.ncbi.nlm.nih.gov/ (accessed on 23 February 2022) with the search term “Glycyrrhiza polysaccharides” are shown in Figure 1.

Per our findings, for the first time in 1986, Shi and Yang reported mitogenic activities of LPs on murine spleen cells proliferation, later in 1989, Chang reported that LPs have anti-viral properties [25,26] as a result, it has piqued the interest of academicians to investigate more about its extraction, composition, and effectiveness. In terms of pursuing LPs functions in numerous biochemical processes, structural features and orientation are also critical aspects. Nevertheless, since 2017, LPs potentials are being investigating in pharmaceutical formulations as nanocarrier, formulation modifiers, and biosensor development due to their good thermodynamic profile.

It is the purpose of this paper to review the LPs extraction processes, their compositions, and related potential activities, which can give an insight into developing advanced treatments and strategies for product development.

## 2. Extraction and Purification of Licorice Polysaccharides

### 2.1. Extraction

The plant cell wall matrix is composed of two major chemicals, i.e., hemicellulose and pectin, incorporated with a minor number of structural proteins. The matrix polysaccharides are made up of several polymers that differ depending on the cell type and plant species. Common methods reported for extracting LPs include solvent extraction, ultrasonic-assisted extraction (UAE), microwave-assisted extraction (MAE), enzymatic extraction, and supercritical carbon dioxide (SC-CO_2_). Before starting the extraction process, 4–8 h pretreatment with an organic solvent such as ethylacetate, ethanol, petroleum ether, or a combination of ethanol and petroleum is used to remove the surface lipids.

The solvent extraction method is the most popular method. LPs are either neutral or acidic, so they can be extracted by using solvents such as water, ethyl (methyl) alcohol, or dilute alkali. Herbal plants are crushed into small pieces and subjected to the solvent for hours under a certain temperature. This method has the advantages of simplicity and affordability in cost, but it has drawbacks such as long extraction time, low yields, and high working temperature.

On the other hand, a comparatively efficient method is the UAE, which is based on the combination of ultrasonic cavitation, thermal, and mechanical effects. Ultrasonic cavitation produces a strong physical effect inside the plant cell that breaks the plant cell wall, and the thermal effect makes the material dispersion. The temperature rises to promote the dissolution of the active ingredients, and the mechanical effect makes the medium particles vibrate to strengthen and enhance the diffusion and mass transfer. The UAE method has the advantages of high efficiency, saving energy, and quick operation. Ultrasonic power, liquid to material ratio, time, and temperature are common optimization indexes of this method, as shown in Table 1.

Apart from solvent extraction methods for LPs extraction, MAE is a simple procedure. It breaks the cell wall, allowing polysaccharides to be extracted quickly. The extraction rate is high, the operation is straightforward, and the process moves quickly. To achieve good selectivity, a small amount of solvent is required. Hai used 400 W microwave for 4 min, and achieved a yield of 3.327%, with material to liquid ratio of 1:30 g/mL. The orthogonal test based on the single factor test revealed that the order of the components’ effects was extraction time > microwave power > solid: liquid [27].

Enzymatic extraction has also attracted more attention. It uses enzymes as the catalyst to destroy plant cell walls and release the active components from the cell under mild conditions. This method effectively maintains the biological stability and potential of active substances. The use of enzymes improves the extraction of water-soluble polysaccharides in a simple and predictable way, but it needs an accurate pH and temperature with a specific enzyme concentration, otherwise, inactivation of enzymes occurs, and yield reduces. Li et al., used 2% of cellulase and pectinase for LPs extraction with a material to liquid ratio of 1:20 g/mL and time of 2 h, achieving yields 10.71% and 8.43%, respectively [28].

A newly developed “green separation technology” is SC-CO_2_ extraction technology. It has the advantages of non-toxicity, no solvent residue, low-temperature treatment, strong selectivity, non-flammability, and safety. The main limitation of this technology is high-cost machinery and operation at >15000 psi. [29,30]. *Inflata* licorice root under treatment of 37.7 Mpa at temp. 62.6 ℃ for 1.38 h gave LPs yield of 7.34% [31].

Recently, Yue reported an ultrasonic-assisted deep eutectic solvent method for extracting LPs and optimized the method using a design of response surface experiment. The extraction rate was 8.31% with a choline chloride-isopropanol system with 40% water, material to liquid ratio of 1:50 g/mL, and the applied ultrasonic power was 250 W for 0.5 h at 39 °C [32].

Considering any method from Table 1, the LPs content in extracted solution varies depending on the extraction time, temperature, and liquid to material ratio, which in turn depends upon the part of the plant used.

### 2.2. Removal of Impurities

The veracity of biological activity depends upon components’ purity, which in turn ensures the quality and safety of the product. After extraction, crude polysaccharides need to be isolated from impurities such as pigments, protein traces, and or inorganic molecules. The method to remove impurities includes alcohol precipitation, macroporous adsorption, sevag method to remove proteins, and preparative chromatography.

Since LPs are insoluble in organic solvents, precipitation with alcohol helps to eliminate impurities. The optimized conditions for LPs were reported as ethanol concentration of 80%, flocculation time of 12 h, and room temperature [33]. The larger in molecular weight, the lower alcohol concentration is needed to precipitate the polysaccharide. Therefore, fractional precipitation with alcohol would produce LPs fractions with different molecular weights. This process can reduce the burden in the later gel permeation chromatography (GPC) step.

Macroporous resins are used to separate and enhance biologically active chemicals from a variety of natural products. The crude polysaccharide from the leaf often has more pigments. In the de-pigmentation process, macroporous resins are often reported. The polysaccharide will not retain on a very hydrophobic frame of macroporous resin. Yijun has used HPD-722 resin with 50% alcohol to increase the purity of extracted crude LPs polysaccharides up to 44.01% [34].

The content of protein in crude polysaccharide depends on the part of the plant and varied with the planting location. Crude LPs have protein in the range of 7~10%, but Wittschier reported the presence of protein content up to 18% in raw LPs [35]. The sevag technique (reagent CHCl_3_/BuOH = 4:1 *v*/*v*) is often used to remove protein impurities. Wei used the sevag method following purification with AB-8 macroporous resin, and then finally with preparative chromatography, the *inflata* LPs with a purity of 91% were achieved [36].

### 2.3. Purification of LPs

Crude LPs contain polysaccharides of different molecular weights, monosaccharide compositions, and linkage types. Therefore, a refining procedure is needed. The methods of purifications and separation for LPs included anion exchange, GPC, microporous resin, and affinity chromatography. Anion exchange chromatography, especially with diethylaminoethyl (DEAE), is the prior choice for purifying LPs. GPC is used next to the DEAE procedure, to refine and/or desalt the fractions.

LPs from the root of *ural* licorice were purified with DEAE-52 and followed by Sephadex G-100 column chromatography, which gave three distinct polysaccharides. Zhang analyzed the *ural* licorice from Ningxia (China) has the Mw of 1.0160 × 10^4^, 1.1680 × 10^4,^ and 1.3360 × 10^4^ Da, and the ratios of glucose (Glc) were 23.4%, 14%, and 1.13%, respectively [37]. Wang has analyzed *ural* licorice from Gansu (China), using ultrasonic-assisted extraction, and found three polysaccharides of Mw 4.513 × 10^3^, 1.378 × 10^5,^ and 2.084 × 10^5^ Da, respectively, with glucose and galactose (Gal) as the main components [38].

To sum up, the methods of extraction and purification of polysaccharides plays a vital role in end product achievement [39], and some examples are summarized in ta-ble 1 to understand the gain of monosaccharides proportions in given licorice polysaccharides fraction.

**Table 1:** Examples of extraction and purification methods.

	Method of Extraction and Solvent Used	Extraction Conditions Liquid: Material (mL/g), Temp., and Yield	Purification via	LPs Purity %	Mw	Fraction Name	Comments	Ref.
*Ural***licorice** Location Extracted part								
Ningxia, China, Root	Water	9, 1 h, 80 °C	DEAE-52 and Sephadex G-100 column chromatography	85.23	10160 Da	GUPs-1	Highest proportions of *Glc 23.4, *Gal 25.18 and *Ara 8.32.	[37]
				84.16	11680 Da	GUPs-2	Protein 6.12%, *Glc 14, *Gal 25.67 and *Ara17.54	
				83.24	13360 Da	GUPs-3	High protein association 28.01%, *Gal 22.04 and *Ara 31.44. this fraction is arabinogalactan protein.	
Gansu, China Root	Water Ultrasound power 600 W	13, 1.42 h, 70 °C, 4.32%	DEAE-52 and Sephadex G-100 column chromatography		4.513 × 10^3^ Da	GPs1	Mainly consist of *Glc 56.08, *Gal 23.97	[38]
					1.378 × 10^5^ Da	GPs2	*Glc 66.42, *Gal 19.12 and *Ara 16.6	
					2.084 × 10^5^ Da	GPs3	Mainly consist of *Glc 48.88 and *Gal 19.89	
Gansu, China Root	Water	11, 2.33 h, 80 °C, 22.31%				GP	Surface Response Method applied optimized extraction process. Gives high yield.	[40]
Xinjiang, China Root	Water	15, 2 h, 99 °C, 16.41%	DEAE cellulose-32 column chromatography and sephadex G-100 column			GUPII	*Glc 5.85, *Gal 3.01	[41]
Xinjiang, China Seed	Water	30, 2 h, 90 °C, 8.1%				GUP	Protein 10.07%, *Man 1.02, *Glc 0.22, *Gal 1.0, *Xyl 0.22, TH structure.	[42]
Zhenjiang, China. Root	Water	20, 3.5 h, 90 °C, 5.67%	DEAE-52 column chromatography and SephadexG-200 gel column chromatography	98.58	294373 Da	GUP1	High proportion of *Ara 37.83, *Gal 18.96, *Man 3.32, uronic acid 12.2%, *GlcUA 17.03, TH structure	[43]
				98.39	17416 Da	GUP2	Highest proportion of *Glc 213.54, *Ara 15.51, uronic acid 1.66%, no TH structure	
*Glabra* **licorice** Location Extracted part								
Karakalpakstan, Uzbekistan Root	Water	30, 3 h, 90 90 °C	DEAE-Cellulose-52 column and Sephadex G-100 column	98.49	3.87 × 10^5^ Da	GPN	*Glc 98.03, protein 1.32%, TH structure	[44]
Xinjiang, China, Leaf	Water	15, 2 h, 120 °C	DEAE-Sepharose fast flow column chromatography	87.46		GP1	*Ara 38.7, *Gal 31.7, *Glc 13.7, *Man 8.1, and *Rha 7.8	[45]
				74.47		GP2		
				52.59		GP3		
Xinjiang, China, Seed	Water	30, 2 h, 90 °C, 8.45%				GGP	Protein 8.73%, *Man 1.22, *Gal 1.0, *Glc 0.24, *Rha 0.22, TH structure	[42]
Hilden, Germany Root	Water	5, [20 h, 8 °C] ×3, 2.5%	DEAE Sephacel column	81		RPS	Protein 18.5%, *GlcUA 18.8, *Glc 16.2, Gal 14.9, *Ara 11.5, *Man 6.92, *Rha 6.9	[35]
*Inflata* **licorice** Loca tion Extracted part								
Aksu, Xinjiang China. Root	Alkaline 5% NaOH	n.m, 2 h, 50 °C	DEAE-cellulose and Sephadex G-150	93.29	2.89 × 10^6^ Da	AGP	Highest proportion of *Glc 3.05, *Xyl 2.85, and *Ara 2.33, TH structure.	[46]
Aksu, Xinjiang	Water	3, 2 h, 80 °C, 4.315	HPLC and Sephadex G-200	n.m	1.96 × 10^6^ Da	GIBP	This fraction contained protein 8.14%, *Glc 4.048, TH structure.	[47]
n.m, Root	Water	10, 6 h, 100 °C, 0.21	DEAE Sepharose Fast Flow and Sepharose CL-6B gel filtration chromatography	94.05	3.3 × 10^5^ Da	GIP1	Highest proportion of *Glc 8.10.	[48]
Aksu, Xinjiang China. Root	Water	3 h, 100 °C	DEAE-52 ion exchange chromatographic column, Sepharose Cl -6B Agarose gel column		2 × 10^6^ Da	GiP2	Highest proportion of *Glc 11.7, homogenous polysaccharide	[49]
					2.1 × 10^7^ Da	GiP3	Highest proportion of *Gal 18,	[50]
n.m, Root	Water	21, 1.38 h, 93 °C, 10.48	DEAE-52 and Sephadex G-75 column chromatography			GPS	Optimized method for extraction	[51]
n.m, Cotyledon	Water, Ultrasonic power 80 W	70, 0.5 h, 90 °C, 7.72%	DEAE-52 and Sephadex G-75 column chromatography	92.685		GPS	Each part needs specific conditions	[52]
n.m, Hypocotyl	Water, Ultrasonic power 80 W	40, 0.3 h, 80 °C, 7.49%		86.424				
Xinjiang China, seed	Water	30, 2 h, 90 °C, 7.83				GIP	Protein 7.4%, *Man 0.97, TH structure.	[42]

n.m: not mentioned. TH: triple helix. * is Highest Proportion of Monosaccharides in Molar Ratio.

It was found that LPs content in the root of *inflata licorice* was the highest, *glabra licorice* was second, and *uralensis* *licorice* was the lowest, even after the same cultivation environment was provided. While the proportion of Man:GalUA:Glc:Gal:Ara was 1.0:6.7:8.0:1.5:2.5, 1.0:0.4:7.7:2.3:1.0, 1.0:6.3:2.2:0.9:1.7, respectively [53,54,55].

Rozi determined the LPs content in seeds of licorice (Xinjiang, China). All the three species have xylose, mannose, glucose, galactose in ratio of 0.22:1.20:0.22:1(*ural*), 0.27:0.97:0.31:1(*inflata*), 0.22:1.22:0.24:1(*glabra*) [42].

The effect of season on the accumulation of polysaccharides in *ural* licorice was also studied. The polysaccharide content of spring collected was much greater than that of autumn collected from the two-year-old plant [54].

The bioactive ingredients content of cultured licorice has been found significantly lower than that of wild licorice [56,57] which can also be speculated for polysaccharides content.

In general, it is challenging to obtain pure polysaccharides by using a single approach; hence, a combination of procedures is required to accomplish effective LPs separation. The scope and order of application of each method should be considered. Even after purification, the LPs are still a group of molecules, like polymers. The purified fractions may have similar composition, but a different degree of polymerization. It is the reality for all polysaccharides. The elucidation of the molecular features of polysaccharides necessitates the development of new methodologies and resources, but unfortunately, there is no breakthrough in purification technologies in the past 40 years.

## 3. Characterization of LPs

Natural polysaccharide functions and behaviors are frequently impacted by their molecular features, which include monosaccharide content, glycosidic bond, configuration, sequence, molecular weight (Mw), and chain conformation. The structural analysis of carbohydrates is generally regarded as one of the most difficult undertakings in glycosciences.

Due to the structural diversity and variability of natural polysaccharides, accessing the fine structure in all hierarchies is extremely difficult [58]. Glycosidic bonds can form in a variety of places, resulting in varied connectivities. Additionally, each glycosidic connection creates a new stereocentre, which can be α- or β-configured [59]. Moreover, in solution form, glucopyranose exists in the so-called mutarotation equilibrium of both α-anomeric form (34%) and β–anomeric form (66%), and they are capable of interconversion between these forms. Nuclear magnetic resonance spectroscopy (NMR) technology has been used to characterize polysaccharides, including anomeric topologies, sequences, and patterns of glycosidic connections.

### 3.1. Primary Structure Analysis with NMR

Anomeric configuration and glycosidic linkage define the biological activities and predictable interactions of polysaccharides with other molecules. NMR analysis gives a plethora of information on an entity. However, due to the large molecular structure of polysaccharides, substantial overlapping of the peaks makes the analysis challenging, particularly for heteropolysaccharides. Generally, in the ^1^H-NMR spectra of polysaccharides, the end-substrate signal appears between 4.5 and 5.6 ppm. The end-matrix is placed comparatively downfield compared to protons at other places. The accompanying signal peaks (two couplings) are well separated, corresponding to a single hydrogen doublet. In addition, the α-anomeric proton resonates further downfield (5.1 ppm) from the β–anomeric proton (4.5 ppm) making these two anomers distinguishable by ^1^H-NMR even at low field, and all other protons in the polysaccharide have peaks at 3.5–4.6 ppm.

**Figure 2:**
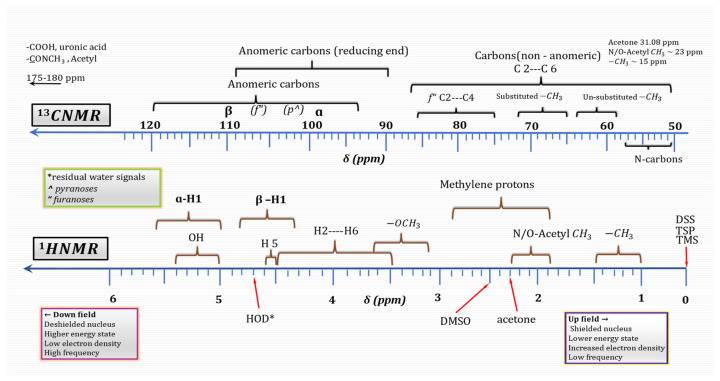
Carbohydrate predictable regions in ^1^H and ^13^C NMR spectra chemical shift assignments [60,61,62].

^13^C signals are not generally dependent on the number of atoms that generate each one, comparable to ^1^H spectroscopy. As a result, the most essential pieces of evidence supplied by a carbon spectrum are the number of distinct signals and their chemical changes. The majority of polysaccharide peaks in the ^13^C-NMR spectra appear between 50 and 120 ppm. However, substitutions can greatly move the signals downfield, for instance, 7–10 ppm downfield signals appear due to O-alkylation and <3 ppm due to O-acylation. Anomeric carbons can be found at 90–110 pmm. In contrast to the ^1^H-NMR spectrum, the ^13^C-NMR spectrum has a much wider range of displacement values and few signals overlap. Figure 2 summarizes the general distribution of carbon and hydrogen chemical shifts in polysaccharide analysis, based on our experience and reported in the literature.

Using ^13^C NMR, Tomoda identified the presence of acidic polysaccharides consisting of six residues in *ural* licorice. Signals at *δ* 178.26 ppm and *δ* 21.75 ppm indicated the presence of O-acetyl group and O-methyl at *δ* 57.04 ppm as carboxylic acid methyl ester [63]. Glycosidic bond in AGP was identified by Zhang, and the resonances of seven anomeric hydrogens were found in the region of *δ* 4.5–5.9 ppm confirms the α-type glycoside and β-type glycoside, and an additional peak at *δ* 1.20 ppm*,* using ^1^H NMR. While ^13^C NMR spectra showed seven anomeric carbons resonances in 90–110 ppm, and an additional peak at 16.68 ppm, the additional peak in both spectra shows the presence of methyl group of rhamnose [46]. However, the methyl group resonances were found at 1.03 ppm and 1.05 ppm of rhamnose residue. These chemical shift difference in the methyl group is due to the presence of protein (chemical shifts at 1.1–3.3 ppm) providing a “shielding effect” (σ) to the protons of methyl groups. The protons of aromatic amino acids were assigned to the broad peaks at *δ* 6.8–7.7 ppm. The chemical shifts showed that *glabra* licorice contained a large number of arabinogalactans [64]. While spectra of GUPIII showed chemical shifts at δ 1.12–1.19 ppm, indicated the presence of methyl groups of rhamnose residues, and *δ* 2.02 ppm and *δ* 1.99 ppm were associated with acetyl groups binding at O-2 and O-3 of Gal*p*A, respectively, in ^13^C NMR spectrum, the chemical shift at 175 ppm showed that the α-D-GalpA was partly methyl esterified [65]. Table 2 summarizes LP analysis through NMR, which would be helpful in future LP NMR analysis.

NMR resonances may be broad due to the polysaccharide’s high Mw. High temperature and high magnetic fields help to obtain more resolved spectra. Partial hydrolysis or ultrasonication can be used to reduce its weight for better structure elucidation. Curve deconvolution is frequently used to resolve overlapping signals during the data manipulation stage.

Apart from NMR, some chemical technologies, such as monosaccharide analysis with liquid chromatography after hydrolysis and linkage pattern analysis with GC –MS after methylation, hydrolysis, acylation, etc., are required for the complete elucidation of LP’s structure [68,69].

### 3.2. Secondary Structure Analysis

Secondary structure means that how a polymer looks, the term “conformation” is used to define this property. Biological molecules’ specific conformation is crucial because it affects biochemical activities. A biopolymer may be a spherical, coil, rod, single helix, double helix, or triple helix. Basically, the glycosidic bond, both inter- and intramolecular H-bonding, is responsible for achieving and maintaining the specific shape of a polysaccharide. The three-dimensional shape can be achieved in liquid or as a solid, which can be analyzed by differential scanning calorimetry (DSC), atomic force microscopy (AFM), x-ray diffraction (XRD), circular dichroism (CD), and Congo red test. Currently, the most popular analysis technique to identify polysaccharide conformation is Congo red test.

Polysaccharides form complexes with Congo red in the dilute alkaline solution, but when the concentration of NaOH increases, the maximum absorption wavelength increases (from λmax 486 nm to longer λmax). Due to the high density of charges supplied along the strands, the triple helix dissociates, resulting in electrostatic charge repulsion between the strands that make up the helix, and causing changes in the triple helix structure to double helix and then random coil or single-chain conformation [70]. The λmax also decreases then, because Congo dye cannot bind to a random coil, resulting in a graph. Mutaillifu identified the triple helix (TH) structure of GPN in *glabra* licorice by the variations in the λmax of Congo red and GPN complexes with varying NaOH concentrations (0–0.50 mol/L). The highest UV–Vis absorption wavelength of the sample increased from 486 nm in water to 493 nm in 0.1 M NaOH solution, indicating the presence of a triple-helical structure in GPN. Similarly, several research groups also confirmed the presence of the TH structure of LPs [42,44,46,47]. The significance of the TH structure of LPs will be discussed as a reference in Section 5 of this paper.

## 4. Pharmacological Activities

LPs have multiple biological activities, such as immunoregulatory, anticancer, antioxidant, antiviral properties, anti-apoptotic, and antidiabetic. LPs are investigated in vivo*,* animal feed additives, vaccinations, and veterinary medications at certain doses. The bioactivities of LPs are discussed in a well-organized paper with details [71]. This review summarized the data in Table 3 and discussed more details on dose and structure-activity to understand the mechanism of action on given LPs.

Studies on LPs with low molecular weights have proven that they could be immunomodulatory and anticancer compounds. They are non-cytotoxic, suppress tumor growth, increase immune organ weight and index, activate immune cells and stimulate secretion of anti-inflammatory cytokines, and inhibit secretion of pro-inflammatory cytokines [71]. These polysaccharides, therefore, have potential use in cancer immunotherapy.

Li et al. have found that in vitro, at a concentration of 2 mg/mL of *ural* LPs alleviated the myotube atrophy induced by the co-culture system of C26 colon cancer cells and RAW264.7 macrophages via inhibiting the STAT3 signaling pathway [72]. Similarly, an in vivo study on mice has confirmed *ural* LP immunomodulatory effect at a dose of 100 mg/mL by increasing the body weight, serum IL-2, CD4^+^/CD8^+,^ and the activity of natural killer cells [73].

A study on human hepatocellular carcinoma cells and their mechanism via apoptosis assay, real-time PCR, and Western blot analysis on imprinting control region mice showed that at a dose of 1250 μg/mL *glabra* LPs can suppress tumor growth by influencing the P53/PI3K/AKT pathway [74].

Acidic homogenous LPs extracted from *Inflata*, having a higher concentration of galactose, the backbone consist of 1,4-linked-α-Gal*p*A and 1,2-linked-α-Rha*p* while side chains 1,5-linked-α-Ara*f* and α-Gal*p,* which are mainly liked at O-4 of α-Rha*f,* showed immune-enhancing properties [75].

LPs also show antioxidant activity. It was observed through an increase in glutathione peroxidase, lactate dehydrogenase, and a decrease in malondialdehyde in lactating pigs by adding 2.5% *ural* LPs in their diet [76]. Cheng investigated *ural* LPs for anti-viral adjuvant properties and found that 100 mg/kg of Mw 1–2 × 10^5^ Da LPs have a significant effect on foot and mouth disease (FMD) immunized mice, splenic lymphocytes, the serum IL-2, and IL-6 levels had significantly improved [77].

Different monosaccharide composition and orientation produce variances in structure and thus account for the considerable differences in bioactivity. Both α- and β-glycosidic linkages were found in *ural* licorice, structural analysis showed that GUP-1 and GUP-2 from *ural* licorice have different structural information, but both contained traces of nitrogen, and Mw 2.94373 × 10^5^ Da and 1.7416 × 10^4^ Da, respectively. The content of glucuronic acid in GUP-1 and GUP-2 was 12.2% and 1.66%, respectively. GUP-1 has a triple helix structure and mainly consisted of glucuronic acid, while GUP-2 is composed of glucose, and has a higher ratio of 1→3 glycosidic bonds. Only GUP-2 had the strongest ability to reduce the blood uric acid in the acute hyperuricemic rat model [43]. GPs1 from *ural* licorice (Mw 4.513 × 10^3^ Da) has a flaky pore structure and a large number of dispersions. It presents as a small amount of smaller globular aggregates. It has a powerful scavenging effect on DPPH^•^ and ^•^O_2_^−^ radical, compared to GPs2 (1.378 × 10^5^ Da) and GPs3 (2.084 × 10^5^ Da) [38]. *Ural* licorice obtained from Gansu contains a higher proportion of glucose which showed antioxidant activity, and antibacterial against Escherichia coli and Klebsiella resistant strains [78].

It was concluded that *glabra* LPs have more antioxidant capabilities than *ural* LPs [44]. Moreover, high Mw *glabra* LPs were found to inhibit proliferation of human oral cancer cells by inducing apoptosis via mitochondrial pathway [48].

Neutral LPs obtained from *Inflata* having 1,3-linked-α-Gal*p* as a backbone and a higher ratio of galactose also showed immune activities [50]. GCI from *glabra* leaf contains a higher ratio of arabinose having linkage position as →2)Ara*f*-(1→, →6)-Gal*p*-(1→ and 6)-Gla*p*-(1→ showed antioxidant properties [45].

*Inflata* LPs, of Mw 1.96 × 10^6^ Da can inhibit 64.77% α-glucosidase at 6 mg/mL, and an antioxidant effect at 3 mg/mL was observed by Pan et al., conducting α-glucosidase (1 U/mL), DPPH^•^, ^•^OH, ^•^O_2_^−^, ABTS^+•^ scavenging activity [47].

LPs are biologically active compounds that can be used as antibiotics or as a preventative medication in the promotion of growth and regulation of human health and in animal husbandry. It can boost the immune system, inhibit tumor growth, and boost antioxidant capabilities.

However, the toxicological and antagonistic effects of LPs are lacking. Comparative studies with other TCM plant polysaccharides need to be evaluated to obtain a firmer foundation for LPs pharmacological applications in clinical settings.

**Table 3:** Medicinal properties of licorice polysaccharides and mechanism of action.

Species	Experimental	Model	Mechanism	Concentration	Ref.
*Ural* **licorice**	Acute hyperuricemia was induced by oral administration of hypoxanthine and intraperitoneal injection of potassium oxonate.	In vivo	Inhibition of XO.	300 mg/kg	[43]
	Scavenging ^•^OH, ^•^O_2_^−^, 1,1- DPPH^•^ on oil.	In vitro	Antioxidant.		[79]
	Mononuclear cells isolated from cord blood collected under axenic condition.	In vitro	Immunomodulator effect on differentiation, maturation, and immune activity of DC.	400 µg/mL	[80]
	Growth performance, immune organ indexes, immunologic functions.	In vivo	↑Body weight, serum IL-2, CD4^+^/CD8^+^ and the activity of NK cells.	100 mg/mL	[73]
	Myotube atrophy model of C2C12 cells based on co-culture system of C26 colon cancer cellsand RAW264. 7 macrophages.	In vitro	Down-reg. of p-STAT3/STAT3 via inhibiting RAW264. 7 macrophages.	2 mg/mL	[72]
	36 COPD cases of phlegm dampness.	In vivo	Immune regulation.		[81]
	Suckling piglets’ diet.	In vivo	↑ GPx, ↑ LDH, ↓ MDA.	2.5% LPs in diet	[76]
	Production of TNF-α and the expression of TNF-α mRNA using BALB/C mice.	In vivo	↑ TNF-α, ↑ TNF-α mRNA (antitumor).	200 µg/mL	[82]
	Rat liver tumor.	In vivo	↑ TNF-α.	30 mg/kg	[83]
	Pentylenetetrazol kindled epileptic rats.	In vivo	Down-reg. of P2 × 7 receptor and NF-кB protein expression in hippocampus, ↑MDA, IL-18, TNF-α.	50 mg/kg	[84]
	Fresh human blood.	In vitro	Promote γδ T cells, ↑cytokines secretion.	100 mg/L	[85]
	Colon microorganism of broilers.	In vivo	Proliferation of bifidobacteria and lactobacillus, inhibition of Escherichia coli and Salmonella.	1000 mg/kg	[86]
	Mouse peritoneal macrophages.	In vivo	Expression of iNOS mRNA and Generation of NO and iNOS → ↑Synthesis of NO.	400 µg/mL	[87]
	Immunized FMD Mouse spleen.	In vivo	Indirect activate DNA polymerase, ↑DNA synthesis, ↑Lymphocytes, IL-2, IL-6.	100 mg/kg (1–2 × 10^5^ Da)	[77]
	In feed, broilers body weight.	In vivo	↑IGF-1 gene expression.	900 mg/kg	[88]
	CT 26 tumor-bearing mice immune organ indices, immune cell population, and serum cytokine levels.	In vivo	Immunomodulator activity via activation of CD4^+^ and CD8^+^ immune cells, increasing IL 2, IL 6, IL 7 levels.	500 mg/kg (under 1 × 10^4^ Da)	[89]
	TCDD-induced hepatotoxicity in Jian carp fish.	In vivo	↓ ALT, AST, LDH, and AKP; ↑ Alb, CAT, GPx, T-AOC and SOD; inhibit MDA; enhanced expression of cytochrome P4501A (CYP1A), AHR2 and ARNT2 mRNA.	1 g/kg	[90]
*Glabra* **licorice**	DPPH^•^, ABTS^+•^, and ^•^OH, scavenging essay.	In vitro	Antioxidant.	0.186 mg/mL	[44]
	Citric acid-induced cough efforts in guinea pigs.	In vivo	Antitussive action (immunomodulator).	50 mg/kg	[64]
	Kunming mice fed high-fat diet.	In vivo	↑spleen lymphocytes, ↓serum IgA, IgG and IgM, ↑SOD, CAT, GSH-Px, and TAOC, ↓MDA.	100 mg/kg	[91]
	Human hepatocellular carcinoma cells and its mechanism via apoptosis assay, real-time PCR, and Western blot analysis on imprinting control region mice.	In vivo	Tumor suppressor through influencing P53/PI3K/AKT pathway.	1250 μg/mL	[74]
*Inflata* **licorice**	DPPH^•^, ^•^OH, and mouse splenocyte.	In vitro	Antioxidant, ↑ proliferation of splenocytes.	100 µg/mL	[92]
	Apoptosis of human oral cancer SCC-25 cell line.	In vitro	Down-reg. of Blc-2, up-reg. of Bax, release of cytochrome c, activate the initiator caspase-9 and effector caspases-3, cleaves PARP.	200 μg/mL	[48]
	α-glucosidase (1 U/mL), DPPH^•^, ^•^OH, ^•^O_2_^−^, ABTS^•+^.	In vitro	Inhibition of α-glucosidase and antioxidant.	64.77% α-glucosidase inhibits at 6 mg/mL, antioxidant at 3 mg/mL	[47]

Note: Up-reg.: Up-regulation; Down-reg.: Down-regulation; ↑: increase; ↓: decrease; →: leads to. Abbreviations: ABTS: 2,2’-azino-bis(3-ethylbenzothiazoline-6-sulfonic acid); AHR2: Aryl hydrocarbon receptor 2; AKP: Alkaline phosphatase; Alb: Albumin; ALT: Alanine aminotransferase; AST: Aspartate aminotransferase; ARNT2: Aryl hydrocarbon receptor nuclear translocator 2; Blc-2: B-cell lymphoma 2; Bax: Blc-2 Associated X-protein; CD4^+^/CD8^+^: Cluster of differentiation; CAT: Catalase; COPD: Chronic Obstructive Pulmonary Disease; DC: Dentritic cells; ^•^DPPH: 1,1-diphenyl-2-picrylhydrazyl; FMD: Foot and Mouth Disease; GPx or GSH-Px: Glutathione Peroxidase; IGF-1: Insulin like Growth Factor-1; Ig A/G/M: Immunoglobulin A/G/M; iNOS: induced Nitric Oxide synthase; IL: Interleukins; LDH: Lactate dehydrogenase; MDA: Malondialdehyde; NF-кB: Nuclear Factor-kappa-B cell; NK: Natural killer; PI3K/AKT: Phosphatidylinositol 3-kinase; PARP: poly(ADP-ribose) polymerase; p-STAT3/STAT3: phosphorylated-Signal Transducer and Activator of Transcription 3; SOD: Superoxide dismutase; TNF-α: Tumor Necrosis Factor-alpha; γδ T: Gamma delta T cells; TCDD: 2,3,7,8-tetrachlorodibenzo-*p*-dioxin; T-AOC: Total antioxidant capacity; XO: Xanthine oxidase.

## 5. Uses of LPs as Biopolymer

Polysaccharides are abundantly found in nature. From the perspectives of structure, thermodynamics, and postulated biological values, the molecular genesis of polysaccharide functions are examined. Their low-cost manufacturing, bioactivity, non-toxicity, biocompatibility, biodegradability, and water-solubility properties make them the most appealing and prospective biomaterials and nanocarriers. Nevertheless, polysaccharides feature a large number of reactive functional groups on their backbone, i.e., -OH, -COOH, NH_2_, which can be easily derivable and contribute to their structural and functional diversity. This is evidenced by their widespread use as excipients in traditional pharmaceutical formulations as well as in other clinical settings.

### 5.1. Triple Helix Structure and Thermodynamics of LPs

**Figure 3:**
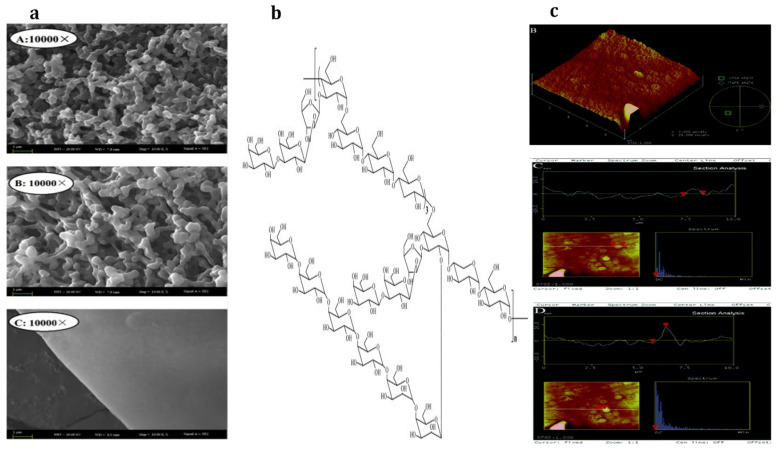
Triple helix structure of LPs. (**a**) SEM micrographs of polysaccharides from the seeds of the [A] *ural,* [B] *inflata,* and [C] *glabra* licorice at 10,000× magnification [42], (**b**) proposed structure of polysaccharide GIBP from *inflata licorice* [47], (**c**) FAM micrographs of the polysaccharide from the root of *ural* licorice [B] angular view, and [C and D] section analysis [94].

LPs from roots and seeds are found to have a triple-helical (TH) structure (as shown in Figure 3), which is particularly intriguing because of its proclivity for forming higher complex structures. The helical configuration allows for the effective packing of molecules and consequently significant energy storage capacities. In addition to this, the TH structure polysaccharides proved far more strength and stability apart from their role as anti-cancerous and anti-tumorous effect and these activities still depend on the ratio between mass and the number of branches in the triple helix polysaccharides.

The presence of protein in crude polysaccharides enhances the properties through “associative interaction” because they interact both via physical (hydrogen bonding, electrostatic or van der Waals) bonding as well as chemical bonding (polar, non-polar, ionic) in a system. Such entities have a wide application in pharmaceutical-based formulations and food product development. Additionally, they are considered, “Generally Recognized as Safe” (GRAS) [42,44,47,93,94,95].

The thermodynamic properties of a polymer are an important factor for its incorporation with other materials as these properties enable them to interact with additional supramolecular interaction by chemical modification or copolymerization. In the context of the solvent media, one must additionally consider enthalpic and entropic modifications. Functional polymers that are responsive to various external stimuli, such as physical, chemical, and biological signals, are a promising study topic [96].

Wang extracted acidic polysaccharides from *ural* licorice and examine their thermodynamic properties. They found that ultrasonic-assisted, water extracted LPs have 3.68% protein, 2.94% sulfated radicals, and a uronic acid content of 35.71%. LPs were branched, without starch and non-reducing sugars. The LPs could dissolve in both hot and cold water but were unable in organic solvents. Their thermodynamic data shows that LPs are non-Newtonian fluids, which means that the viscosity varies with shear stress. At a power of 4.585 mW/mg, the melting point of GP was raised from 501.2 to 509.9 °C, followed by heat release, and the enthalpy change of the exothermic reaction was 11.989 kJ/g, such properties are presented by complex polymers, and they have good thermal stability [97]. However, no uronic acid was detected in purified GPN from the *glabra* licorice but had a starch content of 4.56%, protein of 1.32%, and total sugar of 98.49% [44].

Pectic polysaccharides are widely used in food processing, *ural* and *inflata* licorice have active pieces of pectic polysaccharides, having neutral glycosyl side chains, uronic acid unit, and rhamnogalacturonan core were all implicated in the expression of biological activity as well [66,98].

Galactomannans are well-known biopolymers, and high Mw galactomannans are desirable as emulsifiers, stabilizers, thickeners, and sorbents for heavy metal ions in a variety of industries [99]. The results of a study showed that the seeds of *ural* licorice contain galactomannan, whose major backbone is comprised of (1→4) bound β-D-mannopyranose residues substituted at C-6 with single α-D-galactopyranose residues. Freshly matured seeds have a polymer with a fine structure having high viscosity [η] 1193.1 mg/g and higher Mw 1.379 × 10^6^ Da, higher (55.9%) proportion of disubstituted (Gal) Man–Man (Gal) unit, and a significantly smaller fraction of monosubstituted (Gal)Man–Man and Man–Man (Gal) units (18.4%). Overwintered seed galactomannan has a relatively lower viscosity, lower Mw 8.77 × 10^5^ Da, and a larger proportion (32.5%) of monosubstituted units [67].

**Figure 4:**
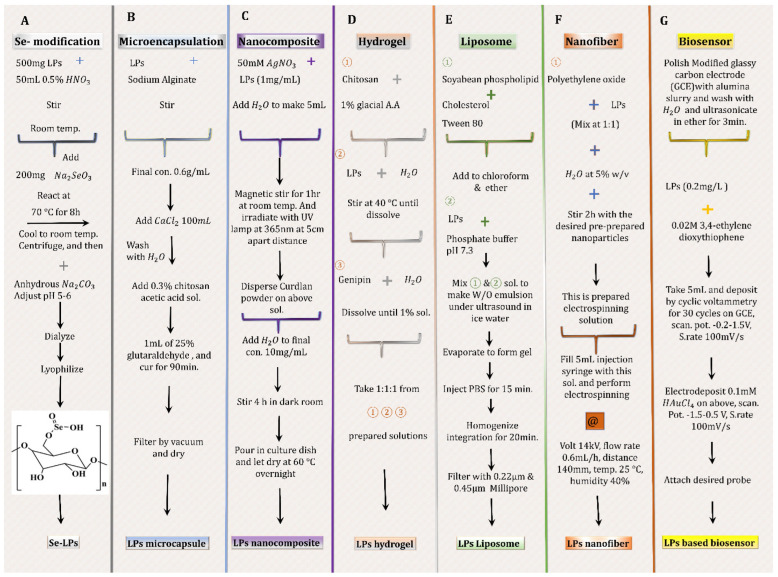
Some methods of formulation using licorice polysaccharides. Formulation method, quantities and conditions are retrieved from: (**A**)**:** selenium modification [101]; (**B**): LPs microencapsulation [40]; (**C**): LPs nanocomposite [105]; (**D**): LPs based hydrogel [106]; (**E**): LPs liposome [107]; (**F**): LPs nanofiber [109]; (**G**): LPs based biosensor [111].

**Figure 5:**
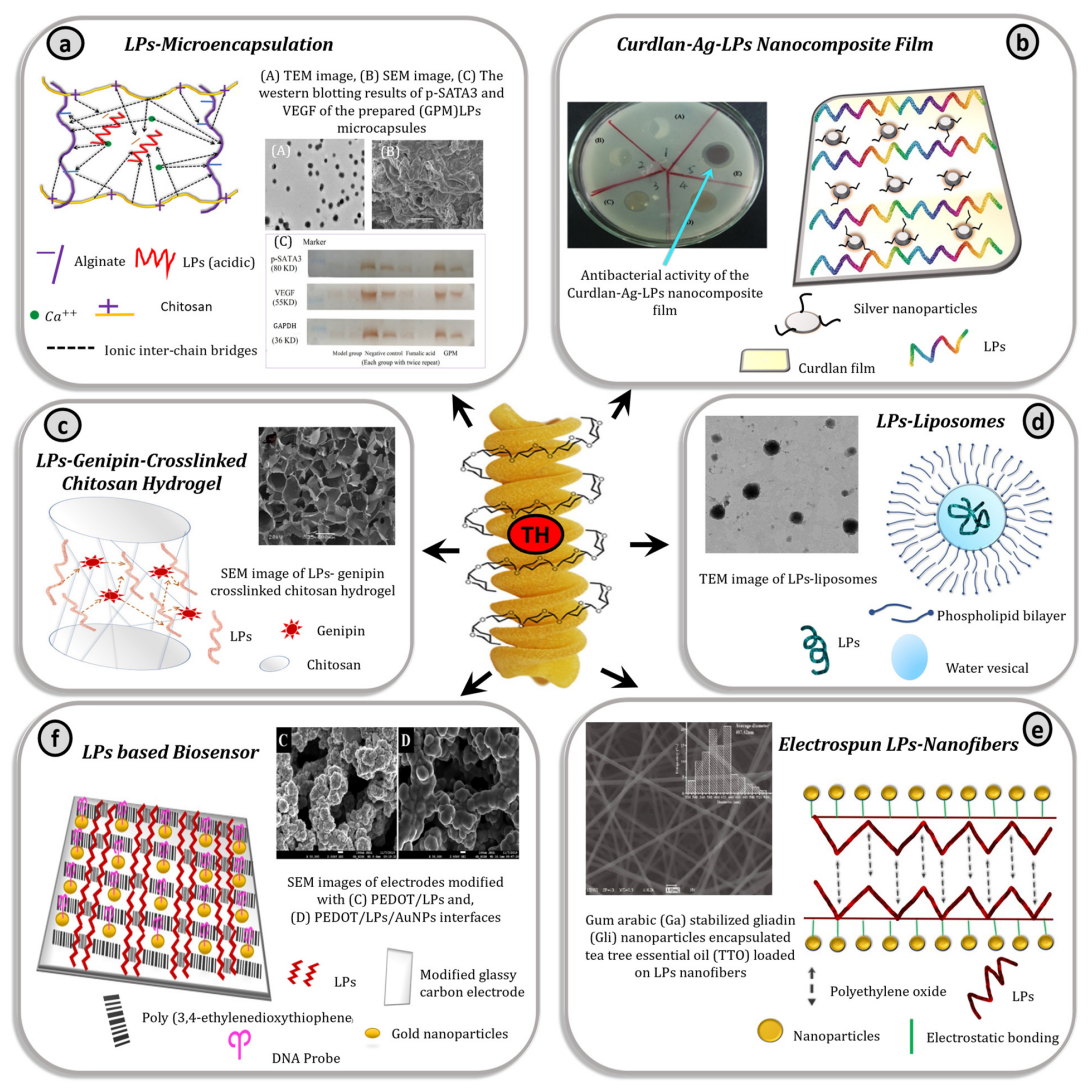
Corresponding results LPs-based formulations. (**a**) LPs based microencapsulation (speculated diagram) and results A, B, and C obtained from [40], (**b**) LPs based nanocomposite film (diagrammatic concept adopted), and the experimental results obtained from [105], (**c**) LPs based hydrogel (speculated diagram) and result obtained from [106] (**d**) LPs based Liposome (speculated diagram), and experimental result obtained from [107], (**e**) LPs based electrospun nanofiber (speculated diagram), and experimental result obtained from [109], (**f**) LPs based biosensor (diagrammatic concept adopted), and results obtained from [111]; TH; diagrammatic concept of triple helix structure of LPs.

Below are some of the recent advancements in the use of LPs, which can be attributed to their TH structure, associated thermal stability, and inherent bioactivity. Their methods of preparations are illustrated in Figure 4 and corresponding results are shown in Figure 5.

### 5.2. Selenium Modification

Selenium (Se) is a trace mineral that is vital to human health. It modulates the expression of at least 30 seleno-proteins in the human body, such as iodothyronine deiodinases, glutathione peroxidases, and thioredoxin reductases. These selenoenzymes can regulate physiological functions in the human body by serving as preventing cancer, antioxidants, enhancement of sperm production and quality, regulating thyroid hormone metabolism, and immune system function.

Natural selenium polysaccharides from plants, mushrooms, and bacteria have already been isolated. However, they are not commonly found in nature and the Se content is also insufficient. Synthetic selenium polysaccharides and selenium nanoparticles coated with polysaccharides have been made using several methods in recent research [100]. Lian formulated selenized *ural* licorice polysaccharide (Se-GUP) using the nitric acid-sodium selenite method. The analysis through FT-IR showed that it exists in O-Se-O form. The resulted Se-GUP has reduced Mw but having 1.33 mg/g selenium, particle size reduced by 49.5% and zeta potential value was −33.8 mV. These properties contribute to formulation dispersion and intestinal absorption, but they might cause thermal instability. Se-GUP has been shown in studies to offer a number of great biological features for usage as medicine or adjuvant [101].

Zhu formulated Se-GUP and studied its anti-inflammatory activity and immunoregulatory effects. Inflammation-induced mice were treated with low, medium, and high doses of Se-GUP and GUP for 10 days. The results showed that each dose of Se-GUP could significantly inhibit the auricle swelling induced by xylene, at 300 mg/kg dose it significantly reduced the increase in capillary permeability induced by acetic acid and inhibited the inflammatory cytokine TNF-α and IL-1 β at 200 mg/kg dose, relative to GUP alone. However, in vitro and in vivo, the formulated Se-GUP comparatively showed greater antioxidant activity [102].

Teng investigated the activity of Se-GUP for acute liver injury, induced by CCl_4_ in Kunming mice, the results showed that it has a hepatoprotective effect via scavenging free radicals [103].

The enhanced bacteriostatic effect was observed in vitro on resistant strains of S.aureus and E.coli, providing a certain additive effect of Ceftiofur sodium or Kanamycin in combination with Se-GUP. The immunization test in vivo, after 7 days of treatment with Se-GUP showed an increase in the content of IgG, IL-1, and IL-2 in the serum of mice to varying degrees, the medium (200 mg/kg) and high (300 mg/kg) dose groups of Se-GUP have a significant immune regulation effect [104].

### 5.3. Microencapsulation

Microencapsulation is the process of isolating an active substance from its surroundings by encapsulating it in a capsule with a diameter varying from 3–800µm. The substance then escapes through the capsule wall in a variety of ways, such as breakage, dissolution, melting, or dispersion. In the manufacture of wound healing materials, tissue engineering materials such as alginate, cellulose, hyaluronic acid, chitin, and chitosan have been widely used. As the description of tissue engineering material, soluble LPs and chitosan exerted strong antibacterial effects, showing that the LPs/alginate gel was suitable to be used as a wound dressing.

By microencapsulating of cross-linking sodium alginate, a calcium chloride-LPs complex was generated as a new biomedical material. The entrapment rate of 59.92% was achieved, by the combination of 2% sodium alginate, 0.3% chitosan, 3% calcium chloride, and 0.6 g/mL of *ural* LPs. The physical properties of LP-based microcapsules showed adhesiveness, hardness, and elasticity were 0.0372 ± 0.00599, 2.0 ± 0.157, and 0.18 ± 0.000258, respectively. The weight loss occurred at 65% at 109.7 °C and decomposition at 150 °C and 550 °C which is attributive to the condensation of the system. The swelling rate reached equilibrium in 36 min, could absorb water, and peeled off, having a 16-day in vitro degradation rate; indicating a good capability of satisfying the microcapsules formulation. The wound healing property was assessed using a rat model. The results showed that it can remarkably activate the expression of p-STAT3 and VEGF proteins, the transcription of VEGF mRNA and miRNA-21 genes, and microvessels in wounds increased and hence promoted healing [40].

### 5.4. Nanocomposites

Nanocomposites are hybrid materials made at the nanometric scale (<100 nm) by combining polymers with inorganic solids. Because of their small size and high surface-to-volume ratio, nanocomposites have chemical and physical properties that distinguish them from their bulk counterparts.

Silver nanoparticles (AgNPs) are frequently reported as antibacterial material. Most of the formulated AgNPs rely on using certain reducing agents and polymers for cross-linking. Reducing agents such as hydrazine or borohydride are toxic, leading researchers to look for an alternative to toxic chemicals. In the creation of soft nanomaterials, polysaccharides are a green alternative to synthetic polymers. Cai prepared nanocomposite (20–50 nm) with acidic polysaccharides from *glabra* licorice incorporated Ag and integrated into a biopolymeric film of curdlan, which showed high wetting property and maintenance of its shape after 16 h in PBS, and had a clear antibacterial effect, providing an attractive template for the development of novel antibacterial biomaterials. The incorporated LPs acts both as reducing agent as well as stabilizing agent [82,105].

### 5.5. Hydrogel

Hydrogels are hydrophilic polymeric networks that are held together by a range of physiological or chemical crosslinks in three dimensions. Its attractive properties are low stiffness and water retaining capability. Polysaccharides have gained a lot of attention among the many polymers that can be used to generate hydrogels. LPs were mixed with chitosan and crosslinked with genipin. The results showed that swelling ratios ranged from 986% to 1677%, with stiffness values ranging from 777% to 1792% Pa. The inclusion of LPs lowered the mechanical strength of the hydrogel and slowed their gelation and breakdown. Hydrogel made from 1% genipin, 3% chitosan, and 4% LPs were found to have the best bactericidal and fibroblast reproduction boosting properties. It showed good swelling and disintegration rate over time and was more suited to effective recovery of chronic wound infection. These findings suggest a new approach for improving the antibacterial property and cytocompatibility of chitosan hydrogels containing water-soluble active LPs [106].

### 5.6. LPs Liposomes

One of the popular nano-drug delivery systems is “Liposomes”. Nanoliposomes are nanometric cargos with a diameter of less than 200 nm and a larger surface area than normal liposomes. They are spherical-shaped one- or two-layer structures. The inside cavity is made up of hydrophilic molecules aimed at water suspension and the bilayer membrane has lipophilic ends made up of phospholipids. Furthermore, on the basis of the lipid bilayer, liposomes are divided into two major groups. Liposomes with a single lipid bilayer are referred to as unilamellar, whereas those with two or more are referred to as multilamellar.

LPs from *ural* licorice were explored in unilamellar liposome formulation for the first time, by Wu, using the reverse phase evaporation method. The optimized ratio of soybean phosphatide to LPs was 24:1, temp. 46 °C and ultrasound time 16 min. The achieved liposome was spherical, uniform in size 136.4 nm, and had an entrapment efficiency of 78.33 ± 0.25%. In vivo, high and medium doses of liposome could significantly enhance the maturation and proliferation of bone marrow-derived dendritic cells in chicken which in turn stimulated T-cells and cytokines IL-2, IFN-*γ,* and IL-10 secretion. The results showed that liposomes improved immune-enhancing activity compared to LPs alone [107]. LP liposomes can be further decorated with imaging probes, ligands, or drugs/protein entrapment to widen their applications.

### 5.7. Nanofibers

Particles having a size range from 50–1000 nm are called “Nanofibers”. The incorporation of bioactive chemicals into polymer scaffolds for steady and prolonged drug release has emerged as an appealing area of research. The polysaccharides-based nanofibers production via electrospinning is difficult but it gives the advantage of high porosity, superb surface functioning, and the huge surface area needed for environmental applications [108].

Cai designed gum arabic (Ga) stabilized gliadin (Gli) nanoparticles encapsulated tea tree essential oil (TTO) and loaded them on LPs nanofibers. The addition of gum arabic to Gli nanoparticles increases their stability and TTO embedding efficiency and *ural* LPs increased the release efficiency. The diameter of nanofibers was an average of 407 nm and showed the novel application of meat preservatives against Salmonella typhimurium. During the 5 days storage conditions, the use of nanofibers inhibited the bacterial growth and slowed the lipid oxidation process in pork and chicken meat by 98.52% and 97.86%, respectively [109]. Such nanofibers have practical applications in the food sector providing food loss and inhibiting food-borne diseases.

### 5.8. Biosensor

Biosensors are multifunctional assemblies made up of matrix-bound bioactive compounds that are responsible for specific species recognition and thus perform a biochemical assay. The main problem with biosensors is that after some time their surface becomes electrodeposited with nonspecific protein adsorption and leading to false-negative results. Polysaccharides are recognized as antifouling compounds and gained popularity in biosensors production. Polysaccharides are non-conductive in nature but doping with conductive polymer could make them useful in designing an antifouling and sensitive biosensor [110]. Wang developed a low fouling, label-free biosensor based on LPs doping with Poly (3,4-ethylenedioxythiophene)-AuNPs. This biosensor performance was evaluated by microRNA detection, which has demonstrated good sensing capabilities with detection limit as low as 300 fM (range 0.01–10 nM), and high reproducibility, showing great potential in the biomedical field [111]. These results suggest that LPs in industrial and medical fields have prospective technical applications.

### 5.9. Vaccine Adjuvant

In populations that do not respond well to vaccination, adjuvant material is needed to add to the vaccine to boost the immunogenicity of antigens, elicit stronger immune responses, and lower vaccine dosage and production costs [112]. LPs have various pharmacological activities as we have discussed in Section 4. The presence of α-1,4 and α-1,6-linkage [113,114] in polysaccharides can help to enhance the body’s natural immune system as well as stimulate antigen-specific immunity. The linkage type of 1,4 glucopyranose residue was the predominant (77.6%) linkage type in *glabra* LPs [44]. Therefore, it has the potential to be used as adjuvant material for a vaccine. To identify the capability of *ural* LPs as immune boosters against Newcastle disease virus (NDV), Wu has showed that administration of LPs in a vaccinated chicken group could boost their immune response compared to the only vaccinated group [115]. Although this study did only oral administration of LPs which increased the response against the virus, it also gives a clue that LPs can be used as vaccine adjuvant in future formulations. TCM polysaccharides’ potential as vaccine adjuvant is well discussed by [116].

## 6. Conclusions and Perspective

This review summarized the extraction and purification, characterization, pharmacological activities, and formulation applications of licorice polysaccharides. The genus Glycyrrhiza has 29 species and 6 variations but only *Glycyrrhiza uralensis* Fisch, *G. glabra* Licorice, and *G. inflata* Batalin, collectively called licorice, are the most medicinally used and scientifically explored species. They are proven rich in many chemically active components and considered superior to the other species for the corresponding bioactivities.

LPs from the root contain large proportions of glucose, galactose, and arabinose, while leaf and seeds have higher proportions of arabinose, galactose, mannose, and rhamnose along with protein association. The polysaccharides composition of LPs is influenced by physical conditions such as geographic location, growth years, and processing, as well as by extraction and purification methods. The extraction method affects the monosaccharides’ composition, molecular weight, and structures of polysaccharides. Among different extraction methods, ultrasonic-assisted extraction is a cheap and fast method for LPs extraction. However, it must be noted that the triple helix structure of LPs might be destroyed if extraction occurs at high power. The triple helix structure of LPs is vital to performing its function in formulations. Then, solvent extraction is preferable with optimized temperature.

Considering structural and conformational studies, both primary and secondary structures of LPs are important to evaluate their performance for the intended use. The structure elucidation needs purified fractions and sophisticated techniques such as NMR and GC-MS. Therefore, few reports investigated the structures of LPs from root, leaf, and seed. LPs from root and seed are reported to have TH structure but an exploration of the leaf is still pending. Both seed and leaf are reported to have high Mw LPs. High Mw LPs are serviceable for food and medicines formulations. Certainly, it is necessary to explore more about composition and structure to obtain benefits from licorice.

LPs have potential as therapeutic agents for treating a wide range of malignant disorders, through significant pathways and at certain doses. Antioxidants and immune regulation are the most reported bioactivities of LPs.

Presently, biomaterial is a hot topic in both medical and industrial fields. LPs are hydrophilic in nature, containing an abundance of -OH and -COOH groups. They are biocompatible and biodegradable, and thus deemed eco-friendly biomaterial. The fundamental disadvantage of LPs could be the inherent property of non-specific recognition and adsorption with plasma proteins, which might lead to selective reticuloendothelial system absorption, and hence reduced bioavailability. However, on the other hand, they have the potential to allow chemical or enzymatic derivatization, providing opportunities to modify and customize for the intended use. Especially, they can be tailored to create a variety of unique bio-nanostructures, as well as used as nanocarriers. Hence, conjugating LPs in pharmaceutical formulations could avoid facing the last-minute ditch challenges. Most of the extracted LPs fractions fall in the ideal Mw (2 × 10^4^ to 2 × 10^5^ Da [117]) of a polymer for formulations, and vaccine adjuvants [118]. LPs-based nanocarriers provide efficiency to the end products which in turn increases the bioavailability. Another advantage of LPs as nanocarriers is that the preparation under an aqueous environment is favorable for the stability of most drugs, especially protein encapsulation, and reduces the deactivation and toxicity.

LPs’ role in these formulations can give rise to a new horizon of producing more “Biosimilar” products and a green alternative to synthetic polymers in the future.

## Figures and Tables

**Table 2:** ^1^H and ^13^C NMR chemical shift assignments of LPs.

Polysaccharide	Conditions Mentioned in the Literature	Anomeric H (ppm)	Anomeric C (ppm)	Residue	Ref.
UA	Ref. standard: 2,2-dimethyl-2-silapentan-5-sulfonat, temp. 303 K		100.26	α-D-galactopyranosyluronic acid	[63]
			101.21	α-L-rhamnopyranose	
			105.31	β-D-glucopyranosyluronic acid	
			105.87	β-D-galactopyranose	
			110.16	α-L-arabinofuranose	
			111.94	α-L-arabinofuranose	
Gi-A1	60~70 mg + 0.5 mL D_2_O, temp.301.1 K		108.25	α-L-Araf-(1→	[66]
			107. 5	→5)-α-L-Araf-(1→	
			100.4	α-D-Glcp-(1→	
			100.34	→4)-α-D-Glcp-(1→	
			100.49	→6)-α-D-Glcp-1→, and →4,6)-α-D-Glcp-(1→	
			99.37	α-D-Galp- (1→, and →4) -α-D-Galp-(1→	
Gi-A3		5.09	107.1	α-L-Araf-(1→	
		5.25	108.2	→5)-α-L-Araf-(1→	
		5.36	100.6	α-D-Galp-(1→	
		5.4	100.5	→3)-α-D-Galp-(1→, →6)-α-D-Galp-(1→, and →3,6)-α-D-Galp-(1→	
		4.97	99.32	→2,4)-α-L-Rhap-(1→	
Gi-B1		5.17	101.1	α-D-GalpA-(1→, and →4) α-D-GalpA (1→4) α-D-GalpA	
		5.15	100.2	→4) α-D-GalpA-(1→2) α-L-Rhap	
		5.24	99.7	→2-α-L-Rhap-(1→	
		5.22	99.7	→2,4-α-L-Rhap-(1→	
		5.11	107.6	α-L-Araf (1→	
		5.08	108.2	→5)-α-LAraf (1→	
		4.98	96.7	α-D-Galp-(1→	
		4.96	96.8	→3) α-D-Galp-(1→	
GUPII	40 mg + 0.5 mL D_2_O, temp. 333 K, 600 MHz	4.98	107.43	α-L-Araf-(1→	[65]
		4.92	107.32	3)-α-L-Rha-(1→	
		5.21	99.82	3)-α-D-Galp-(1→	
		5.92	99.62	α-D-Xylp-(1→	
		4.51	95.62	→4)-α-D-Glcp-(1→	
GUPIII		4.98	107.37	α-L-Araf-(1→	
		4.96	107.31	3)-α-L-Rha-(1→	
AGP	30 mg AGP + 0.5 mL D_2_O, temp. 298 K, 400 MHz	5.05	107.63	→6)- β-D-Glcp-(1→	[46]
		4.59	106.53	→1)- β-D-Glcp	
		5.11	107.03	→5)-α-L-Araf-(1→	
		5.36	101.77	→4)-α-D-Xylp-(1→	
		5.21	98.72	→6)-α-D-Galp(1→	
		4.94	97.70	→3,6)-α-D-Manp-(1→	
		5.14	99.76	→3)-α-L-Rhap(1→	
GIBP	40 mg + 0.6 mL, 298 K, 400 MHz	5.35	99.82	→4)-α-D-Glc-(1→	[47]
		4.91	99.85	→3,6)-α-D-Glc-(1→	
		5.06	99.14	→2,3,6)-α-D-Glc-(1→	
		5.20	109.43	→2)-β-L-Ara-(1→	
		4.59	104.06	α-D-Gal-(1→	
		4.44	103.09	→3)-α-D-Gal-(1→	
		5.11	107.04	β-D-Gal-(1→	
		5.04	107.54	→3)-β-D-Gal-(1→	
		5.17	92.05	→3)-β-D-Man-(1→	
		4.58	95.93	→3)-α-D-Man-(1→	
GPN	40 mg + 0.6 mL, 298 K, 400 MHz	5.39	100.76	→4)-α-Glcp-(1→	[44]
		5.40	100.89	→4)-α-Glcp-(1→	
		4.97	99.74	→ 6, 4)-α-Glcp-(1→	
		4.80	100.81	→1, 3)-α-Glcp(6→	
		4.65	96.92	β-Glcp-(1→	
Gu-1	5% sample in deuterated dimethylsulfoxide, 125.7 MHz		99.29	α-D-galactopyranosyl	[67]
			101.74	4-O-β-D-mannopyranosyl	
			100.98	4,6-Di-O-β-D-mannopyranosyl	
			93.50	α -D-galactopyranoses	
			94.90	β-D-mannopyranosyl	
